# Probiotic supplementation improves the cognitive function and the anxiety-like behaviors in the stressed rats

**DOI:** 10.22038/ijbms.2019.33956.8078

**Published:** 2019-05

**Authors:** Mahsa Hadizadeh, Gholam Ali Hamidi, Mahmoud Salami

**Affiliations:** 1 Physiology Research Center, Kashan University of Medical Sciences, Kashan, Iran

**Keywords:** Anxiety, Learning and memory, Microbiota, Probiotic, Stress

## Abstract

**Objective(s):**

Prenatal stresses increase incidence of neurodevelopmental disorders and influence cognitive abilities. Glucocorticoids are released in stress condition as endpoint activation of hypothalamus-pituitary-adrenal (HPA) axis. Evidence indicates a cross-talk between gut microbiota and brain function. This study assesses the effect of probiotic supplementation on behavioral function

and HPA axis action in stressed rats.

**Materials and Methods:**

The young rats born from dams exposed to noise stress (ST) during third trimester of pregnancy were used. Two groups of stressed animals were received a two-week probiotic supplementation before (pre-ST) and after (post-ST) birth. The time and distance to find hidden platform in Morris water maze were evaluated as spatial memory. Also entry to open arms in elevated plus-maze was considered as anxiety-like behaviors. The serum level of corticosterone was measured as the HPA axis function.

**Results:**

While the stressed rats decreased entries to open arms to one third compared to the controls (CON) the probiotic treatment increased the entries by two times. The ST rats required more time and distance to find the platform than did the CON animals. The pre- and post-ST rats significantly restored the impaired behavior almost near the CON ones. While the serum corticosterone concentration increased by 50% in the ST rats it was reduced to almost normal level in the pre- and post-ST rats.

**Conclusion:**

Our findings confirmed a link between the gut microbiome and probiotics with the behavioral functions and HPA axis. The probiotic treatment favorably affected the stress-dependent behavioral disorders and the interaction between HPA and gut-brain-microbiota axes

## Introduction

Stressful life events can impair digestion, endocrine and immune responses, behavior and cognition (1). Studies have linked maternal stressors with an increased incidence of neurodevelopmental disorders which, in turn, underlie brain development and cognitive abilities. Accordingly, evidence indicates that exposure to adverse events during fetal life has negative effects on brain development leading to psychiatric disorders (2-6).

Sound pollution is known as one of the most common sources of environmental stress in the world (7). Prenatal exposure to noises is known to be harmful to development of fetus and neonate (8). Importantly, many of neurodevelopmental events during fetal life continue on postnatal period. For instance, offspring born from dams deprived of environmental signals display different function of neuronal circuits (9). The synaptic transmission in the neuronal pathway depends on ionotropic N-methyl-D-aspartate receptors (10) in the hippocampus, an area of brain which is crucial for spatial learning and memory. Development of the hippocampus begins during fetal life and continues to the postnatal period (11). Glucocorticoids underlie normal development of brain via cell survival and initiation of terminal maturation, and remodeling of axons and dendrites (12). However, both reduced and elevated glucocorticoid levels deteriorate development and function of brain (13).

It has been demonstrated that through affecting the expression of pro-apoptotic and anti-apoptotic genes (14) reduced level of neurotrophic factors such as brain derived nerve factor (BDNF) (15) and production of oxidative stresses (16, 17) glucocorticoids cause neuronal death and disrupt neurogenesis in the hippocampus. 

Accumulating evidence from animal studies supports the hypothesis that, through affecting inflammation, the hypothalamic–pituitary–adrenal (HPA) axis and neurotransmission the gut microbiota takes a fundamental role in function of the central nervous system (CNS) (18-22). Many diseases including cardiovascular, metabolic, autoimmune, neurodevelopment and psychiatric disorders have been shown to be correlated with gut microbiota dysbiosis (22-25). There is an obvious relationship between HPA axis and gut microbiota under stress condition where germ free animals display an enhanced HPA stress response (26).

Probiotics are defined as live microorganisms which when administered in adequate amounts they have beneficial effects on the host’s body. Experimental evidence indicates that probiotic treatment can normalize behavioral phenotypes in animal anxiety models (27-31). Our recent findings from human studies indicate that the probiotic supplementation positively affect some of behavioral functions and metabolic statuses in the patients with Alzheimer’s diseases (32) and multiple sclerosis (33).

We previously showed that prenatal noise stress effectively deteriorates spatial memory as well as synaptic plasticity (34). Considering the microbiota-gut-brain axis and the potential role of beneficial bacteria in the CNS function this study was design to evaluate how probiotic bacteria underlie behavioral functions in an animal model of prenatal stress. The learning and memory and the anxiety-like behaviors were assessed by Morris water maze and elevated plus-maze, respectively. Also, via measurement of serum level of corticosterone we explored a link between the function of HPA axis and the probiotic supplementation.

## Materials and Methods

***Animal groups***

Female Wistar rats on night were mated with male Wistar rats. The next day, the cage was explored for a vaginal plug, and if found, this day was considered as the first day of pregnancy. After weaning only the male offspring were kept and raised till adulthood. All animals (whether dams or offspring) were fed with normal food regimen (provided by Razi Research Institute, Tehran, Iran). Six groups of rats were used in this study. Number of animals in the experimental groups (n=6, one animal per cage) was adjusted based on statistical formula for determination of appropriate sample size. 

The control (CON), pre-CON and post-CON rats were born from the control dams (having normal pregnancy with no stress). The stressed (ST), pre-ST and post-ST rats were offspring of the dams exposed to the noise stress during the third trimester of pregnancy (see below). The dams of the pre-CON and pre-ST groups were administered with the probiotic supplementation from the first day of pregnancy for 14 days. The post-CON and post-ST rats received the probiotic supplementation from 31^st^ through 45^th^ day of the postnatal age. The CON and ST groups received drinking water as vehicle. 

***Breeding condition***

Thirty-six male Wistar rats at 45 days of postnatal age were used in this study. All animals including pregnant dams and offspring had free access to food and water and kept in 12 hr light/dark cycle (light on at 6:00 am), temperature of 22 ^°^C, and air humidity of 55–60%. Efforts were made to minimize the number of animals used and their suffering. Adequate measures were taken to minimize animal’s pain or discomfort. All experiments were approved by Deputy of Research of Kashan University of Medical Sciences under project number of 9348.

***Noise characteristics***

The broad band traffic noise was recorded in a high-traffic area by a standard recorder (Panasonic RQ-L11) and amplified by a loud speaker (Power 56W). The intensity of the sound was set at a level of 95 dB by a software (Sonar, Cakewalk). This intensity is comparable with the noise level detected in some industrial workplaces. A precision sound level meter (Extech Instruments, MA) was used to monitor the intensity of sound to 95 dB uniformly in the cage. 

***Noise induction***

The stressed pregnant rats were exposed to the white noise at the third trimester of the gestation period. The cages were placed in a reflective Plexiglas chamber (with dimensions: width, 60 cm; length, 60 cm; height, 90 cm). A loud speaker was installed upper left at a distance of 30 cm above the cage. The pregnant animals were exposed to the noise 4 hr/day (8 am–14 pm) (34). It is reported that the serum level of corticosterone is the lowest during this period of time (35). The control pregnant group was kept in the noise chamber for the same period of time without noise exposure.

***Probiotic supplementation***

The probiotic solution was made by a mixture (each 334 mg) of *Lactobacillus acidophilus* (American type culture collection (ATCC) 4356, ~10^10^ CFU/g), *Bifidobacterium lactis* (Dutch chemical company (DSM) 10140, ~10^10^ CFU/g) and *Lactobacillus fermentum* (ATCC 9338, ~10^10^ CFU/g) dissolved in drinking water. Most of previous studies have used *Bifidobacterium* and *Lactobacillus* preparations, and most of them show improving some CNS functions. Doses of 10^9^ and 10^10^ CFU for 2 weeks in animals and 4 weeks in humans have been sufficient to appear measurable effects (36).

The probiotics were obtained from Zist Takhmir Company, Tehran, I R Iran. Different studies have shown that this composition of the probiotics alleviate anxiety-like behaviors, improve spatial memory and decrease corticosterone and adrenocorticotropine hormone (37-39). The probiotic treated dams (from the first day of pregnancy for 14 days) and pups (from 31 through 45 days of postnatal age) were received the probiotic supplementation via intragastric gavage. The animals were received 1 ml solution/day containing a total of ~10^10^ CFU/g of the three probiotic bacteria. The control animals were received the same volume of drinking water. 

***Experimental design***

**Figure 1:**
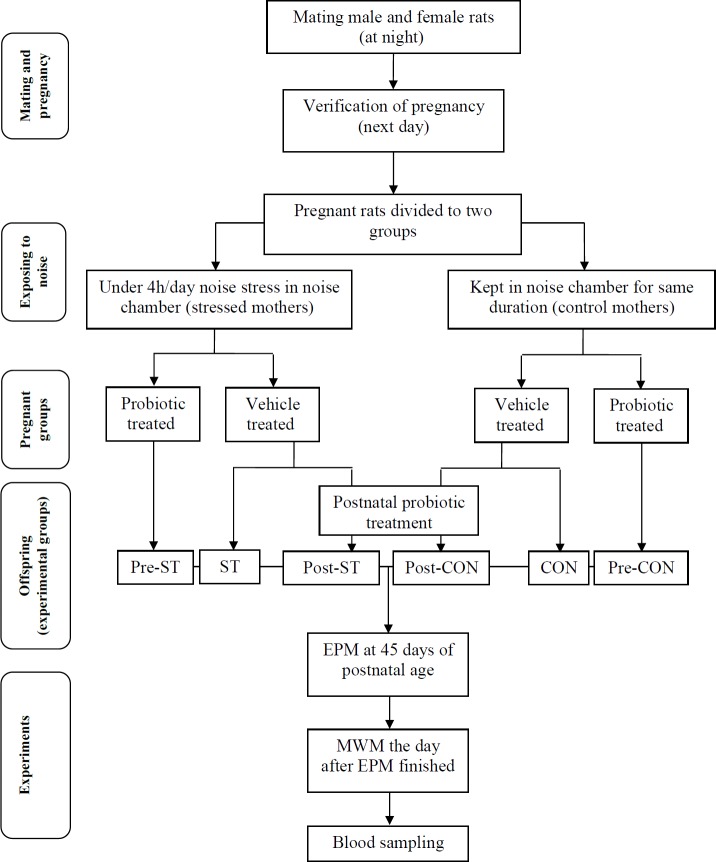
A flow chart for the experimental design

All groups of animals were introduced to the elevated plus-maze at 45 days of postnatal life. Then, the animals were entered the Morris water maze experiments. Blood samples were prepared for plasma level of corticosterone when the behavioral experiments finished (Figure 1). 

***Elevated plus-maze***

The apparatus was consisted of two open and two closed arms (50 cm length and 10 cm width), with opaque walls (25 cm height) and an open roof. Open arms were opposing to each other and intersecting perpendicularly with the opposed closed arms. The maze was elevated at a height of 60 cm from the floor. The testing room was moderately illuminated. At the start of each 5-min trial, animals were placed in the center of the maze facing toward one of the open arms and allowed to explore the maze freely. The number of entries into open arms (OAE) and the time spent in open arms (OAT) were used as indices of anxiety (34). The percent of OAE and OAT were calculated as follows:

OAE% = number of entries into open arms/total number of entries × 100

OAT%= duration spent in open arms (sec) /total navigation time (300 sec) × 100

***Morris water maze test***

Hippocampus-dependent spatial learning and memory was assessed through Morris water maze test as described in the previous work (40). Briefly, a circular galvanized tank (140 cm in diameter × 60 cm in depth) with four quadrants was used. A hidden platform (12 cm in diameter) was submerged 2 cm below the water surface and placed at the midpoint of a fixed quadrant. The water was changed periodically and its temperature was maintained at 22 ^°^C. The walls around the pool were pasted with the surrounding extra-maze visual cues to provide spatial cues for the animal during trials. Four trials/day each for 90 sec followed by a 10 min break were given to each animal for 4 consecutive days. The rats were released into the maze from four different locations (for 4 trials) and allowed to swim for 90 sec. If 

unsuccessful in locating the platform during this time, they were guided to the platform by the researcher. In either case, the rats were allowed to take a rest on platform for 15 s. Each day the test was started from different quadrant for each animal. The performance of rats was tracked and recorded by a video auto-tracking system (RADIAB-7, I.R.Iran). For each trial the time to find the platform (escape latency) and the distance travelled to the platform were measured as learning scores. One day after the last hidden platform test, a spatial probe test was performed to measure the retention of spatial memory. In the probe test the platform was removed and a 30 sec single swimming trial was given to each animal to calculate the time spent in the target quadrant. 

***Serum level of corticosterone***

Blood samples for measuring the serum level of corticosterone were pooled from the animal groups when the experiments finished. Animals were deeply anesthetized and blood was sampled from heart. Then, blood was collected in a sterile tube and centrifuged at 2,500 rpm for 10 min. The serum was obtained and frozen at -20^ o^C until assayed. The concentration of serum corticosterone was measured using a commercially available radioimmunoassay kit (DRG, Germany) according to the manufacturer’s instruction. The manufacturer supplied protocol was implemented. Briefly, the level of corticosterone in the serum samples was single-determined via interpolation from a standard curve derived from six calibrators (ranging from 25 to 1500 ng/ml).

***Statistical analyses***

All the data were plotted as means±SEM. The data pooled from the acquisition phase of Morris water maze and the elevated plus-maze as between-subjects factors and noise exposure as within-subjects factor were analyzed with the one-way repeated measures of variance (ANOVA). Also one-way ANOVA was applied on the corticosterone level of serum with corticosterone and the probe trial in Morris water maze as between-subjects factors and noise exposure as within-subjects factor. For all comparisons a Tukey’s *post hoc* was utilized. *P*<0.05 were considered significant.

## Results

***Assessment of anxiety-like behavior in elevated plus-maze***

Animals in the different groups were introduced to elevated plus-maze to evaluate the anxiety-like behavior. The percent number of entries to and the time percent of stay in the open arms were measured as the indices of anxiety. 

***The percent number of open arms entries***

**Figure 2:**
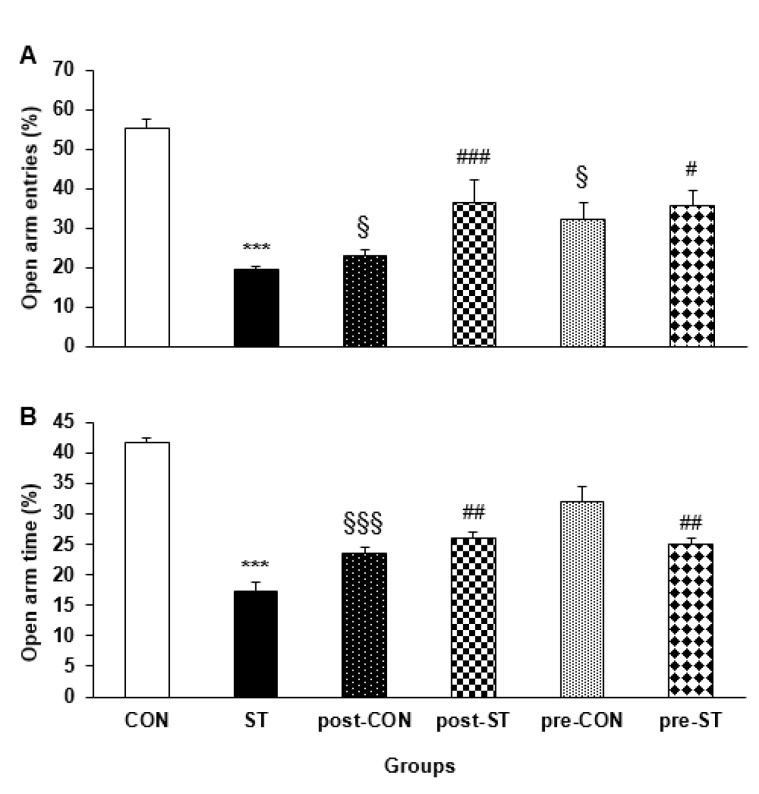
The effect of stress and probiotic treatment on performance of the animals in elevated plus-maze. Values are means±SEM

Analysis of variance indicated that the different groups of animals display a significant difference in the open arm entries (F_5,53_=18.689; *P<*0.0001). The CON rats showed an almost 3 times more entries than their ST counterparts (*P<*0.0001). The probiotic treatment declined the anxiety-like behavior in the pre-ST (*P=*0.02) and post-ST (*P=*0.02) offspring where the number of open arm entries increased (by about two times) in the two groups of stressed rats. The probiotic treated control animals decreased the open arm entries, more pronounced in the pro-CON animals (*P=*0.0001). Figure 2A illustrates that how the noise stress exposure and the probiotic administration affect entry to the open arms. 

***The time percent of stay in open arms entries***

General statistics demonstrated that the testing animal groups differently spent in the open arms of the elevated plus-maze (F_5,53_= 63.353; *P<*0.0001). *Post hoc* analysis indicated that, compared to their CON counterparts, the ST rats stayed for a shorter duration (by about half) in the open arms (*P=*0.0001). The probiotic supplementation considerably improved the maze behavior is the pre-ST (*P=*0.05) or post-ST (*P=*0.0001) animals. Surprisingly, the pro-CON (*P=*0.05) and pre-CON (*P<*0.05) groups had a weaker navigation in the open arms when compared to the CON animals. Figure 2B depicts the effectiveness of noise exposure and probiotic administration on performance of the animals in the open arms.

***Assessment of the spatial learning and memory***

Locating the hidden platform and duration spent in the correct quadrant of the Morris water maze were measured as the learning and the memory consolidation, respectively. 

***The acquisition phase of spatial performance***

The escape latency and the distance traveled by the animals to locate the hidden platform were considered as indices of the task learning. 

***The time elapsed to find the hidden platform***

**Figure 3:**
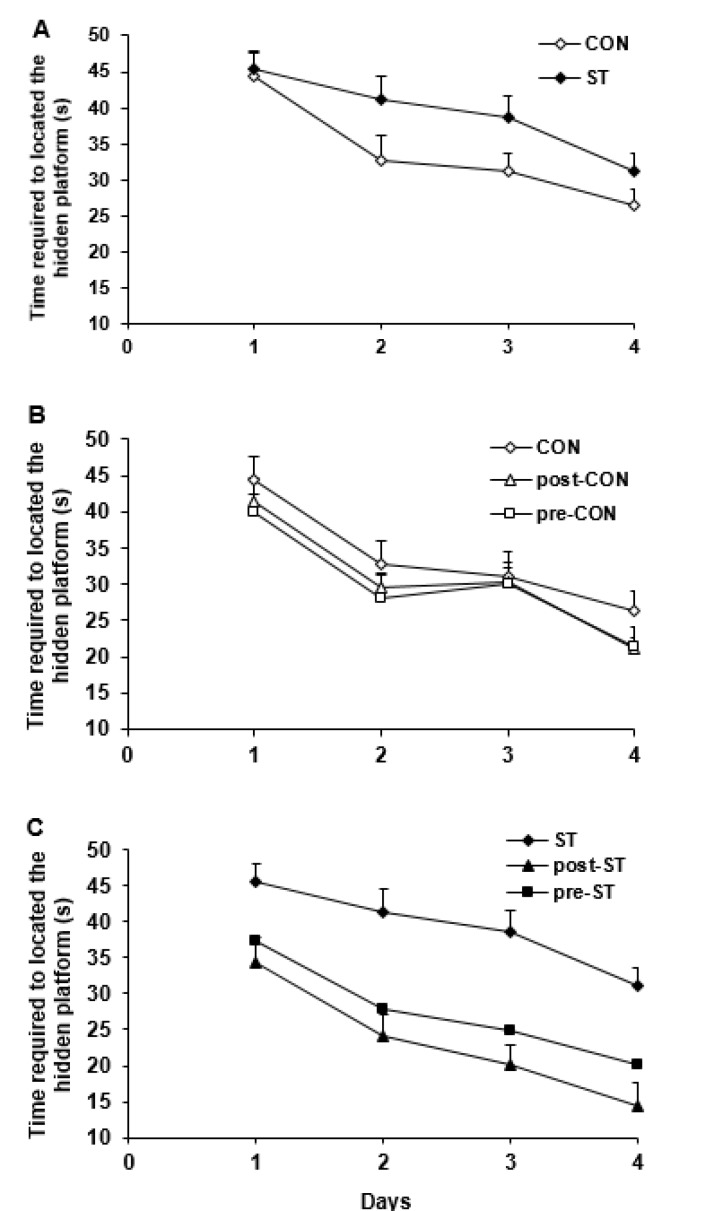
The time required to locate the hidden platform in Morris water maze was used as an index of task learning by the vehicle and probiotic treated control and stressed rats. Values are means±SEM

The data taken from the task learning indicated that the testing groups had a general statistical difference in their behavioral performance (F_5,230_= 8.221; *P<*0.0001). As illustrated in Figure 3A the CON and ST groups displayed the highest and the lowest maze performance, respectively (*P=*0.037, CON vs ST). We found no difference between the behavior of the CON animals with the pre-CON and post-CON group indicating that the probiotic treatment not effectively underlies the spatial navigation in the control rats (Figure 3B). Comparison of the ST group with the stressed animals treated with the probiotic supplements indicated that both pre-ST (*P=*0.0001) and post-ST (*P=*0.0001) rats required a significant shorter time to locate the hidden platform (Figure 3C).

***The distance passed to find the hidden platform***

**Figure 4:**
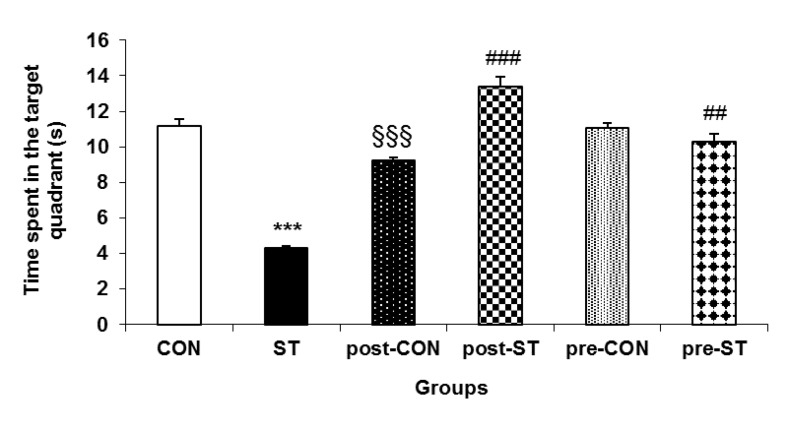
The distance traveled to find the hidden platform was used to evaluate function of the vehicle and probiotic-treated control and stressed groups in the learning of spatial task in the Morris water maze. Values are means±SEM

Considering the distance traveled to find the platform indicated a significant difference in the water maze performance of all groups (F_5,230_= 5.909; *P<*0.0001). The post hoc test indicated that, compared to the CON rats, the ST animals swimmed more distance to find the platform (*P*=0.0001, Figure 4A). The pro-CON and post-CON rats resembled their CON counterparts in the traveled path (Figure 4B). On the other hand, the probiotic supplementation substantially improved the maze steering in the stressed animals so that, in comparison to the ST rats, the pre-ST (*P*=0.05) and post-ST (*P*=0.0001) groups passed a shorter distance to find the hidden platform (Figure 4C). 

***The retrieval probe test***

**Figure 5:**
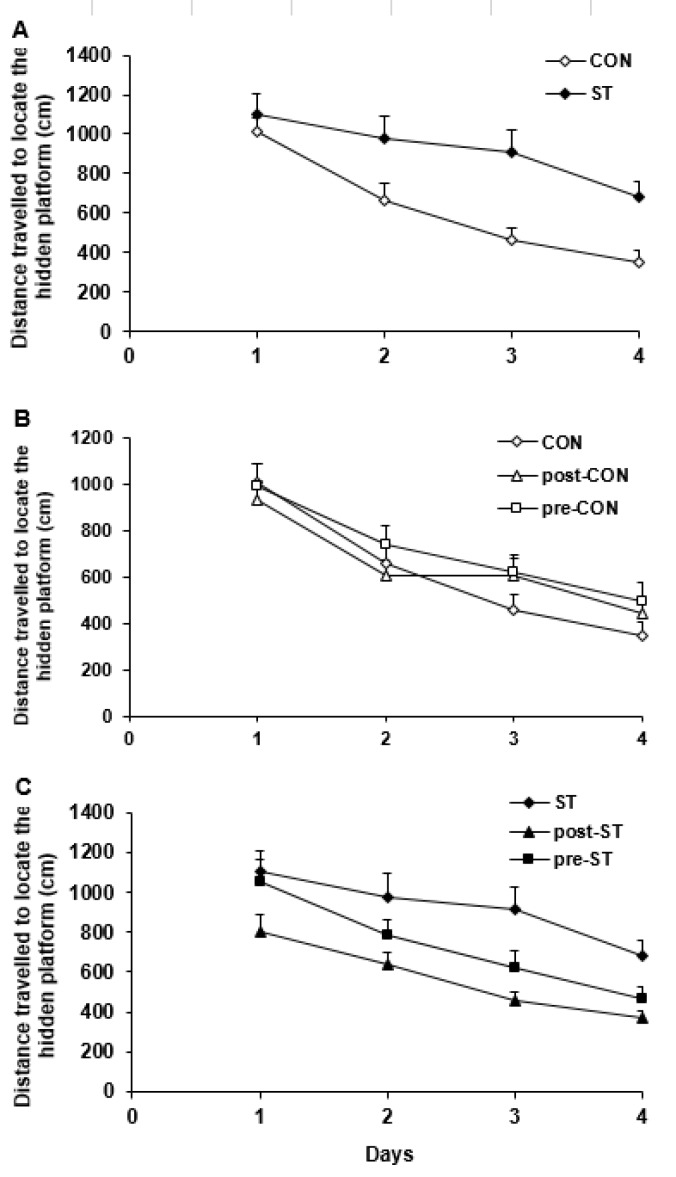
Retrieval test for assessment of memory consolidation in the testing animals. Values are means±SEM. Stress considerably decreased ability of the ST animals in recalling of the target quadrant. While probiotic treatment enhanced the probe trial performance in both pre-ST and post-ST it significantly suppressed the maze performance in the probiotic supplemented controls.

The duration of searching the correct quadrant was considered as index of memory consolidation. Analysis of variance appeared a general difference between performance of the animal groups (F_5,53_= 67.669; *P<*0.0001). Compared to their CON counterparts, the ST rats navigated a shorter duration in the correct quadrant of the maze (*P<*0.0001). Whereas the probiotic administration negatively affected the probe trial in the control animals it had a substantial positive impact on the behavior of the probiotic treated stressed rats where, in comparison to the ST rats, the pre-ST (*P<*0.007) and post-ST (*P<*0.0001) stayed a longer time in the correct quadrant. Figure 5 demonstrates how the prenatal noise stress and the pre or postnatal probiotic administration influence the memory consolidation. 

***Serum concentration of corticosterone***

**Figure 6:**
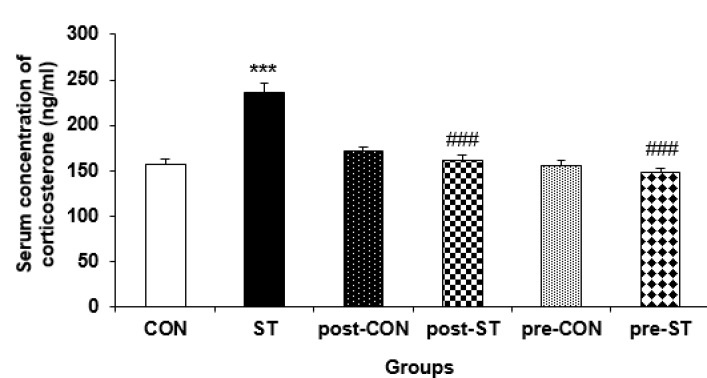
The effect of stress and probiotic treatment on the plasma concentration of corticosterone. Values are means±SEM. The noise exposure significantly increased corticosterone level in the ST rats. The probiotic treatment decreased the stress related hormone in both probiotic-treated ST groups. The probiotic supplementation demonstrated no effect in the control rats

The serum level of corticosterone was measured as an index of HPA axis function. A general significant difference was evident between the corticosterone concentration changes in the control and stressed groups (F_5,4_= 24.726; *P<*0.0001). The noise exposure increased the serum corticosterone (by about 50%) in the ST group compared to their CON counterparts (*P<*0.0001). On the other hand, the probiotic supplementation declined the stressed induced hormone in pre-ST (*P<*0.0001) and post-ST (*P<*0.0001) animals so that no difference was evident between the CON rats and the stressed probiotic treated groups. Pre or postnatal administration of probiotic not efficiently affected the serum concentration of the steroid hormone in the pre-CON and post-CON animals. Figure 6 shows the serum level change of corticosterone in the control and stressed rats.

## Discussion

We found that the prenatal exposure to the noise stress considerably deteriorates behavior of the animals in the elevated plus-maze where the stressed animals decreased either entry to or stay in the open arms. The probiotic supplementation effectively improved the plus-maze performance in the pre- and post-ST rats. We observed no difference between behavior of the control or stressed subjects in response to the probiotic treatment. 

The water maze navigation appeared that the noise exposure substantially suppressed the spatial performance of the stressed animals. The probiotic treatment sufficiently improved the spatial learning and memory in the pre- and post-ST animals. Analogous maze navigation was observed in the pre- and post-CON rats, and in the pre-ST and post-ST animals. 

The current evidence suggests several mechanisms by which probiotics may influence brain function. Probiotics underlie brain biochemistry, by affecting levels of neuromodolators or neurotransmitters such as BDNF, γ-aminobutyric acid (GABA), serotonin and dopamine (28, 41-44), norepinephrine, acetylcholine (45), the vagus and enteric nerves (28, 41)**, **HPA axis (46), and the immune and endocrine systems (47, 48). The present work explores a link between the cognitive and behavioral function of brain with the endpoint of the HPA axis corticosterone. 

It is proved that exposure to chemical, biological or environmental stressors can trigger stress and anxiety responses (23). Human studies have also linked maternal stressors including natural disasters, death of a family member and reported levels of maternal anxiety or depression with an increased incidence of neurodevelopmental disorders including depression, anxiety, schizophrenia, and autism (49, 50). Evidence demonstrates that bacterial presence in the intrauterine environment may underlie the microbiota of infants before birth (51-54). Consequently, during and shortly after birth, infants are exposed to microbes mainly originating from mother. This microbial colonization and subsequent development of the intestinal microbiota in early life is crucial for healthy neurodevelopment and, environmental factors could impact on brain and behavior resulting in brain disorders (23). On the other hand, the gut flora is known to be subject of stress. O’Mahony *et al.* reported that changes in fecal microbiota in early life stress are induced by maternal separation (55). These considerations propose a link between the altered behaviors of the stressed animals with changes in the intestinal microbial colonization. 

pre-ST:stressed rats receiving probiotics from first through 14th day of fetal life; ST: stressed rats; post-CON: control rats receiving probiotics from 31st thorough 45th days of postnatal age; post-ST: stressed rats receiving probiotics from 31st thorough 45th days of postnatal age; CON: control rats; pre-CON: control rats receiving probiotics from first through 14th day of fetal life

A: the ST rats (n=6) were inferior to their control counterpart in the open arm entries. The probiotic supplementation highly improved the anxiety-like behavior of pre-ST (n=6) and post-ST (n=6) groups. *** *P<*0.0001 compared to the CON group, ### P<0.0001 compared to the ST group, # *P<*0.05 compared to the ST group, § *P<*0.05 compared to the CON group

B: the noise stress considerably suppressed the open arm time performance. The probiotic treatment during either prenatal or postnatal life restored the behavior of the stressed rats closer to a normal status. *** *P<*0.0001 compared to the CON group, ## *P<*0.02 compared to the ST group, §§§ *P<*0.0001 compared to the CON group

A: Prenatal exposure to noise stress substantially suppressed learning the maze task where the ST group required a longer time to find the platform than did the CON (n=6) rats (*P<*0.037)

B: The probiotic supplementation not significantly influenced function of the control groups in the water maze

C: Treatment with probiotic bacteria considerably (*P<*0.0001) improved the maze performance in pre-CON (n=6) and post-CON (n=6) so that the both groups located the hidden platform much sooner than their ST counterparts

CON: control rats; ST: stressed rats

A: In comparison to the CON animals, the ST subjects displayed a weaker performance where they passed a longer distance to find the platform (*P<*0.0001)

B: The probiotic supplementation did not affect the water maze navigation in the pre-CON and post-CON rats

C: The probiotic-treated pre-ST (*P<*0.05) and especially post-ST (*P<*0.0001) rats passed a shorter distance to locate the hidden platform

CON: control rats; ST: stressed rats

*** *P<*0.0001 compared to the CON group, §§§ *P<*0.0001 compared to the CON group, ### *P<*0.0001 compared to the ST group, # *P<*0.007 compared to the ST group

CON: control rats; ST: stressed rats

*** *P<*0.0001 compared to the CON group, ### *P<*0.0001 Compared to the ST group

CON: control rats; ST: stressed rats

Growing body of evidence indicate that probiotic treatment substantially influence some neurological capabilities contributed to the intestinal microbiota. Consistent to our results, certain strains of *Lactobacilli* and *Bifidobacteria* either alone or in combination have normalized behavioral phenotypes in animal anxiety models (27-31). The present results are consistent to reports indicating favorable effect of probiotics on the cognitive trends (37, 56). 

We found that the prenatal stress highly increased the serum concentration of corticosterone in the ST rats. We previously proved that the prenatal exposure to noise stress led to impaired spatial memory and deteriorated synaptic transmission in the 2-month old offspring. These impaired brain functions was associated with increased level of plasma corticosterone (49).

Glucocorticoids, as endpoint activation of HPA axis, are required for normal brain development as they initiate terminal maturation, remodeling of axons and dendrites and impact on the cell survival (12). In final days of rat’s fetal life and hours after birth, the levels of glucocorticoids decrease and remain low until approximately two weeks of postnatal age. During this duration response to stress is decreased; what is named as ‘‘stress hyporesponsive period” (57). In humans the period is thought to emerge during infancy and extend throughout childhood (13). Evidence indicates a rational relevancy between the stress-induced cognition defects and increased concentration of the corticosteroid hormone as is reported in the present work. Glucocorticoids that are released under stress condition can activate apoptotic processes and disruption of neurogenesis in hippocampus through increased expression of pro-apoptotic factors and the production of oxidative stress (16, 58), reduced expression of anti-apoptotic (14) and neurotrophic factors such as BDNF (15). These occurrences lead to an impaired learning and memory (59). Further, it has been shown that stress increases glutamate toxicity (60) that gives rise to neuronal apoptosis and dendritic retraction (61). 

The probiotic supplementation decreased the serum level of the stress hormone in the pre- and post-ST animals. The probiotics had no effect on the normal reared rats. Our results indicated that the effect of the intervention was almost similar in all probiotic treated stressed rats; whether during fetal life or after birth. Some other reports are in line with our finding in that HPA axis is programmed by gut microbiome in stress condition and can be influenced by probiotic administration. Sudo demonstrated that germ free mice display an exaggerated HPA stress response (26). Ait-Belgnaoui *et al.* reported that stress-induced changes in the HPA axis are shown to be sensitive to probiotic administration (62).

## Conclusion

Taken together, the present results indicate that exposing to stress during the fetal life disturb the brain related behaviors. The probiotic supplementation, either during fetal life or postnatal period convincingly improves the impaired behavioral functions. Also, the probiotic treatment before and after birth normalize the serum concentration of corticosterone in the stressed rats. Therefore, it seems that prenatal stress displays a strong power on the behavioral reactions and the level of the corticosteroid corticosterone even 45 days of postnatal age. However, the pre and postnatal probiotic treatment is also strong enough to improve the behavioral functions and adjust concentration of the corticosteroid hormone. 

In conclusion, our findings demonstrated that the probiotic administration has a favorable effect on the brain functions linked to the HPA and gut-brain-microbiota axes. However, more investigations are required to warrant clinical significance of probiotics and verify their roles as probable therapeutical tools. 
